# Faculty and Student Interaction in an Online Master’s Course: Survey and Content Analysis

**DOI:** 10.2196/10464

**Published:** 2019-04-04

**Authors:** Christopher Aylwin

**Affiliations:** 1 Imperial College Healthcare NHS Trust London United Kingdom

**Keywords:** online learning, faculty & student interaction, Community of Inquiry, medicine

## Abstract

**Background:**

The provision of online educational courses has soared since the creation of the World Wide Web, with most universities offering some degree of distance-based programs. The social constructivist pedagogy is widely accepted as the framework to provide education, but it largely relies on the face-to-face presence of students and faculty to foster a learning environment. The concern with online courses is that this physical interaction is removed, and therefore learning may be diminished.

**Objective:**

The Community of Inquiry (CoI) is a framework designed to support the educational experience of such courses. This study aims to examine the characteristics of the CoI across the whole of an entirely online master’s course.

**Methods:**

This research used a case study method, using a convergent parallel design to study the interactions described by the CoI model in an online master’s program. The MSc program studied is a postgraduate medical degree for doctors or allied health professionals. Different data sources were used to corroborate this dataset including content analysis of both asynchronous and synchronous discussion forums.

**Results:**

This study found that a CoI can be created within the different learning activities of the course. The discussion forums integral to online courses are a rich source of interaction, with the ability to promote social interaction, teaching presence, and cognitive learning.

**Conclusions:**

The results show that meaningful interaction between faculty and student can be achieved in online courses, which is important to ensure deep learning and reflection.

## Introduction

### Background

Since the creation of the World Wide Web in the early 1990s, the provision of Web-based educational programs has soared. The emergence of digital technology has allowed universities to veer away from traditional teaching methods and offer Web-based courses to both on-campus and off-campus students. In the United States, participation in at least one Web-based course has risen from 9.6% of students in 2002, to 31.6% in 2016, equating to over 6 million students. Of these, 3 million students are taking exclusively Web-based distance courses [1,2].

The rapid expansion and demand of these courses, however, present challenges to educators, both technologically and pedagogically. The emergence of the Web-based course has removed the co-location of student and faculty and with it any interaction engendered by face-to-face physical meetings. A conceptual framework, known as the Community of Inquiry (CoI) was developed by Garrison et al [3] to help support the educational experience of Web-based courses. The CoI assumes that deep learning requires the development of a community and identifies three elements that are essential to form such a community within these courses: social presence, cognitive presence, and teaching presence.

### Community of Inquiry Framework

The CoI framework [3] is a process model of Web-based learning developed to help guide faculty and student interaction and communication to encourage critical thinking, inquiry, and discourse. The CoI framework suggests that deep learning in a computer-mediated distance learning course occurs through the interaction of three core elements: (1) social presence, (2) cognitive presence, and (3) teacher presence. Dewey’s legacy [4] of a collaborative constructivist learning experience argues that a student will develop meaningful and long-lasting understanding of a topic if supported socially, intellectually, and with the guidance of an appropriately knowledgeable instructor.

#### Social Presence

Defined by Garrison et al [3] as “the ability of participants...to project their personal characteristics into the community, thereby presenting themselves to the other participants as real people,” it has been argued that a social presence should be among the first components to be established in a Web-based course to initiate learning [5]. The CoI model identifies three areas within the presence that can help examine ways that students develop social presence: affective expression, open communication, and group cohesion.

#### Cognitive Presence

Garrison et al define cognitive presence as “the extent to which the participants in any particular configuration of a CoI are able to construct meaning through sustained communication” [3]. It is this element that the authors view as the most important to engender success and is heavily influenced by Dewey [6], Kolb [7], and the science of reflective thinking.

The Practical Inquiry model [8] was developed to define the cognitive presence in the CoI and involves a 4-step process that begins with a *triggering event* when the student encounters a problem that requires a resolution. The *exploration* phase involves the search for information, and the *integration* phase links concepts and creates hypotheses. Finally, the *resolution* phase is where the student has tested these and is able to defend or revise them.

#### Teaching Presence

Teaching presence is defined as “the design, facilitation and direction of cognitive and social processes for the purpose of realizing personally meaningful and educationally worthwhile learning outcomes” [9]. Teaching presence is seen as unifying the social and cognitive processes [3] through direction and leadership of the educational experience. There is a considerable body of evidence suggesting that teaching presence is crucial to student learning [10-14] and that it is a significant determinant to student satisfaction and sense of community.

In the CoI framework, there are three categories of teaching presence that have been identified [9]: design and organization, facilitating discourse, and direct instruction.

### Development of the Community of Inquiry Tool

The CoI framework provides a useful theoretical base to research Web-based learning, but most of the literature has focused on single components of the CoI. To address this, a team of collaborators from a range of institutions developed an instrument to measure the three categories of presence [15]. This quantitative tool comprises a 34-item, 5-point Likert scale and aims to measure students’ perceptions of the social, cognitive, and teaching presence in a Web-based course.

Much of the literature on the CoI has focused on examining single aspects of Web-based courses. Studies of the CoI instrument have been undertaken at a higher education level within business courses, and there have been few studies involving CoI within health care. This study examines the faculty and student interaction of the whole taught-component of an entirely Web-based Master of Science (MSc) medical course. It aims to explore the experience of the CoI facilitated in the program by exploring student-faculty interaction.

## Methods

### Setting

This study was carried out at an inner London university with a student enrollment of just under 17,000. The MSc program studied is a postgraduate medical degree for doctors or allied health professionals. Lasting 2 years, the first year comprises the entire taught component, and the second year is set aside solely for the preparation and completion of a dissertation project.

The first year of the program was the focus of this study. In this year, there were 8 taught modules worth 15 credits, each lasting 4 weeks. The program was entirely Web-based, with up to 48 audio-recorded PowerPoint presentations per module, asynchronous case-based discussions, and 8 synchronous discussion sessions per module. Asynchronous discussions allow groups that are separated in time and place to share knowledge by posting and replying to “threads” that are initiated either by students or faculty [16]. Synchronous discussions were Web-hosted conferences led by one member of faculty to which all students were invited to join.

### Participants

The year of study had 22 participants, of whom 3 were from medical professions other than medicine. Each was a graduate from the field of medicine and its allied professions. All were employed full-time, and their experience varied from first-year post-graduate doctors through to established independent practitioners.

### Design

This research used a case study method, with a convergent parallel design to study the interactions described by the CoI model in a Web-based master’s program. A survey designed to measure the aspects of the CoI within a course using only a single data source was thought to be unlikely to complete the aim in sufficient depth. Therefore, different data sources were used to corroborate this dataset including content analysis of both asynchronous and synchronous discussion forums.

### Survey

The survey collected basic demographic data from each student as well as their perceptions of the course based on the CoI framework. The survey was distributed via email and completed approximately 6 months after completion of the first year of the program.

The study used the CoI instrument designed by Arbaugh et al [15]. This consists of a 34-item survey, each consisting of a 5-item Likert scale. It measures perceived cognitive presence, social presence, and teaching presence. Results were analyzed by calculating a composite score for each question based on the mean responses of all respondents. A further subscale score was calculated based on the mean responses to the relevant questions for social, cognitive, and teaching presences.

### Transcript of Discussion Forums

Asynchronous discussion forums were archived from the academic year studied and transcribed anonymously. A sample of 10 discussion transcripts was chosen for analysis. The first and the last discussion were included, and a further 8 transcripts throughout the year were chosen based on the number of postings as well as the subject heading.

One synchronous discussion forum was chosen and fully transcribed and anonymized. The forum consisted of a session lasting 1 hour and 9 minutes, with one member of faculty, and 8 students. The forum was chosen because it occurred in the middle part of the course when it was anticipated that students and faculty had grown more accustomed to the technology and each other, potentially giving a true reflection of the CoI of the course.

### Ethical Approval and Consent

Ethical approval was sought and approved from the researcher’s own institution, and the ethics department of the university hosting the course. Individual “opt-in” consent was sought from each participant for content analysis of anonymized transcribed synchronous and asynchronous discussion forums. In cases where consent was refused, the content of the individual’s posts was removed from analysis.

### Content Analysis

The transcripts of the asynchronous and synchronous discussion forums were analyzed using a coding protocol based on the description of the CoI framework coding protocol template (Table 1), published within the original CoI paper by Garrison et al [3]. The template has been the subject of further research and validated by a number of studies [11,17,18].

**Table 1:** Community of Inquiry (CoI) framework coding protocol template.

CoI presence	Category	Indicators (examples)
Social	Emotional Expression	Emotions, narratives
	Open Communication	Risk-free expression
	Group Cohesion	Encouraging collaboration
Cognitive	Triggering Event	Sense of puzzlement
	Exploration	Information exchange
	Integration	Connecting new ideas
	Resolution	Applying new ideas
Teaching	Instructional Management	Defining and initiating discussion topics
	Building Understanding	Sharing personal meaning
	Direct Instruction	Focussing discussion

## Results

### Basic Demographics

There were 22 students enrolled for the first year of this master’s course. Two students withdrew their participation early during the academic year. Of the 20 remaining students, their basic demographic details are shown in Table 2.

### Survey Responses

The survey presented the participants with some demographic questions, followed by the 34 items of the CoI instrument [15]. Eighteen of the 20 students who completed the first year of the MSc course completed the survey.

Table 3 shows the responses for the survey grouped by presence, with the mean score and standard deviation. The Likert scale consisted of 1 (strongly disagree), 2 (disagree), 3 (neutral), 4 (agree), and 5 (strongly agree).

A mean score of 4.0 in any item equated to an agreement with the statement, and a standard deviation of less than 1.0 suggests stronger agreement [19].

Table 4 shows the calculation of the mean responses per presence, as well as the overall composite mean score. With small standard deviations, this suggests that course participants strongly agreed that the course delivered a social and cognitive presence and agreed that teaching presence was also observed. Overall, the mean composite score from the CoI survey indicates that a CoI was perceived by the participants of the master’s course.

**Table 2:** Student demographics (N=20).

Characteristics		n (%)
**Gender**		
	Male	8 (40)
	Female	12 (60)
**Occupation**		
	Physician	17 (85)
	Nurse	2 (10)
	Other	1 (95)
**Nationality**		
	United Kingdom & Ireland	9 (45)
	Europe	3 (15)
	United States	4 (20)
	Asia	3 (15)
	Africa	1 (5)

### Asynchronous Discussion Analysis

Ten discussion forums were chosen for analysis. Seventeen of the 22 initially enrolled students contributed to the message boards with 165 individual message posts and a total of 18,233 words available for analysis.

The CoI framework provided a coding template [3] that was used to conduct a content analysis of the asynchronous discussion forums. The frequency of each type of coded presence as depicted in Table 1 was counted, giving an overall aggregate description of the 10 discussions, and the group itself.

In total, there were 269 separate codings of presences across the 10 discussion threads. The majority (n=135) were indicators of social presence, followed by 82 instances coded indicating cognitive presence and 52 indicating teaching presence.

**Figure 1:**
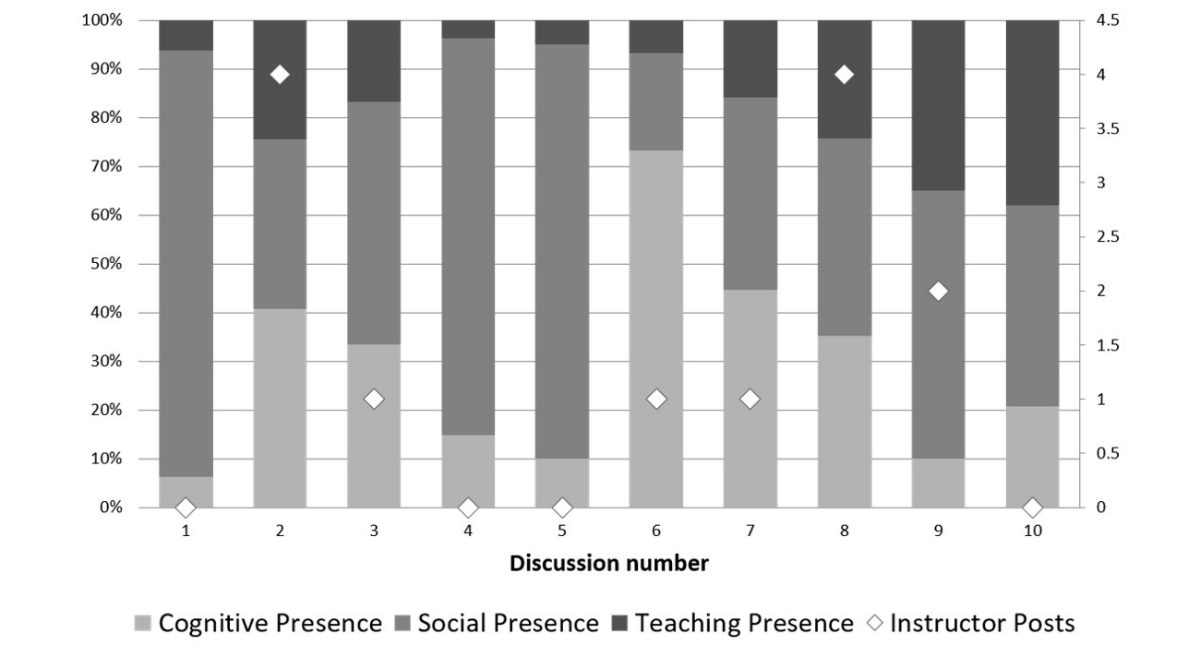
Presences by percent and number of instructor posts.

Instances of social presence were the most prevalent in discussion threads (Figure 1). This is particularly evident when there were no postings from an instructor, as indicated by discussion numbers 1, 5, and 5. When the instructor did post, however, the percentage of teaching presence increased, but the percentage of cognitive presence also appeared to increase.

**Figure 2:**
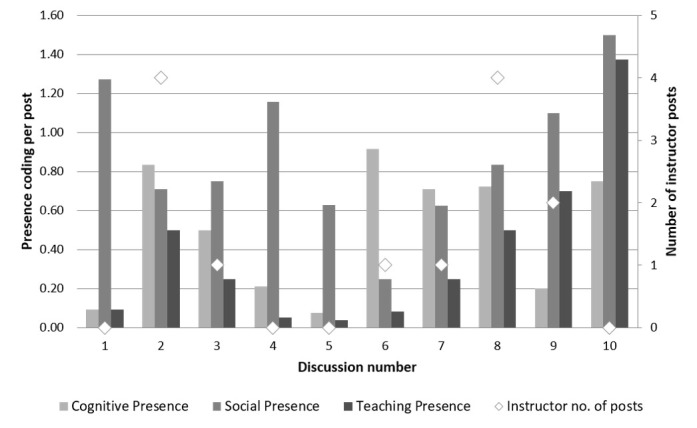
Presences per message post.

**Figure 3:**
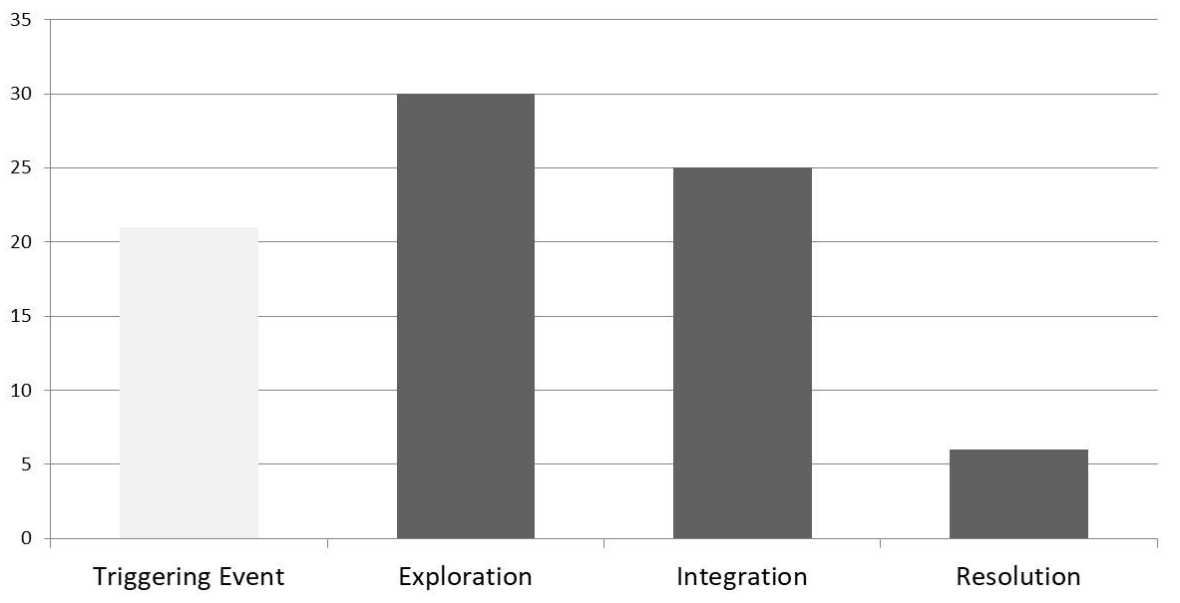
Cognitive presence breakdown for asynchronous discussion.

Figure 2 shows how the individual presence count per message post changed over the course of the 10 threads. Figure 3 is a representation of the proportion of each element of cognitive presence within the coded asynchronous discussions. The majority of instances of cognitive presence are at the lower level of thinking, triggering event and exploration.

**Table 3:** Community of Inquiry survey responses.

Item		Question	Mean (SD)
**Teaching presence**			
	1	Faculty clearly communicated important course topics.	4.2 (0.71)
	2	Faculty clearly communicated important course goals.	3.8 (1.04)
	3	Faculty provided clear instructions on how to participate in course learning activities.	3.8 (0.71)
	4	Faculty clearly communicated important due dates/time frames for learning activities.	3.1 (1.18)
	5	Faculty members were helpful in identifying areas of agreement and disagreement on course topics that helped me learn.	4.1 (0.68)
	6	Faculty members were helpful in guiding the class towards understanding course topics in a way that helped me clarify my thinking.	4.4 (0.51)
	7	The instructor helped keep course participants engaged and participating in productive dialogue.	4.3 (0.59)
	8	Faculty members helped keep the course participants on task in a way that helped me learn.	4.2 (0.55)
	9	Faculty members encouraged course participants to explore new concepts in this course.	4.2 (0.65)
	10	Faculty actions reinforced the development of a sense of community among course participants.	4.3 (0.49)
	11	Faculty members helped focus discussion on relevant issues in a way that helped me learn.	4.3 (0.49)
	12	Faculty members provided feedback that helped me understand my strengths and weaknesses.	3.1 (1.16)
	13	Faculty members provided feedback in a timely fashion.	2.3 (0.71)
**Social presence**			
	14	Getting to know other course participants gave me a sense of belonging in the course.	4.4 (0.85)
	15	I was able to form distinct impressions of some course participants.	4.2 (0.86)
	16	Online or Web-based communication is an excellent medium for social interaction.	3.8 (0.94)
	17	I felt comfortable conversing through the online medium.	3.9 (0.87)
	18	I felt comfortable participating in the course discussions.	4.1 (0.76)
	19	I felt comfortable interacting with other course participants.	4.1 (0.68)
	20	I felt comfortable disagreeing with other course participants while still maintaining a sense of trust.	4.0 (0.59)
	21	I felt that my point of view was acknowledged by other course participants.	4.1 (0.54)
	22	Online discussions help me develop a sense of collaboration.	4.3 (0.57)
**Cognitive presence**			
	23	Problems posed increased my interest in course issues.	4.4 (0.61)
	24	Course activities piqued my curiosity about the subject matter.	4.3 (0.67)
	25	I felt motivated to explore content-related questions.	4.5 (0.51)
	26	I utilized a variety of information sources to explore problems posed in this course.	4.4 (0.51)
	27	Brainstorming and finding relevant information helped me resolve content-related questions.	4.3 (0.57)
	28	Online discussions were valuable in helping me appreciate different perspectives.	4.5 (0.51)
	29	Combining new information helped me answer questions raised in course activities.	4.3 (0.69)
	30	Learning activities helped me construct explanations/solutions.	4.1 (0.68)
	31	Reflection on course content and discussions helped me understand fundamental concepts in this class.	4.7 (0.49)
	32	I can describe ways to test and apply the knowledge created in this course.	4.2 (0.86)
	33	I have developed solutions to course problems that can be applied in practice.	4.4 (0.71)
	34	I can apply the knowledge created in this course to my work or other non–class-related activities.	4.3 (0.57)

**Table 4:** Mean responses for Community of Inquiry (CoI) survey.

CoI presence	Mean (SD)
Teaching presence	3.9 (0.64)
Social presence	4.1 (0.18)
Cognitive presence	4.4 (0.15)
Overall CoI	4.1 (0.46)

### Synchronous Discussion Analysis

One interactive session was chosen. This session was led by one member of faculty, and 8 students logged on for the forum, interacting through video, audio, and text. The transcribed document consisted of 324 individual posts, with 117 posts from the instructor. After removal of the content of the 2 students who did not provide consent, there were 7389 words for analysis.

The same coding protocol was used as for the asynchronous discussions, using the coding template from Garrison et al [3]. The analysis showed a total of 135 presences coded, made up of cognitive presence 47 times, social presence 37 times, and teaching presence 51 times.

Within the synchronous discussion, there were more instances of teaching presence than any other presence. The indicator that was coded most was for direct instruction, followed by explanation of issues (coded by “building understanding”). There were numerous instances of cognitive presence, with similar numbers of each category within the discussion. Within the social presence, there was markedly less group cohesion indicated.

**Figure 4:**
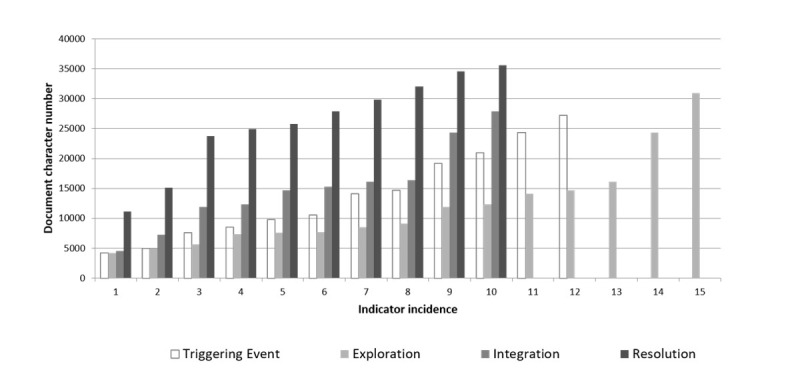
Cognitive presence in synchronous discussion.

**Figure 5:**
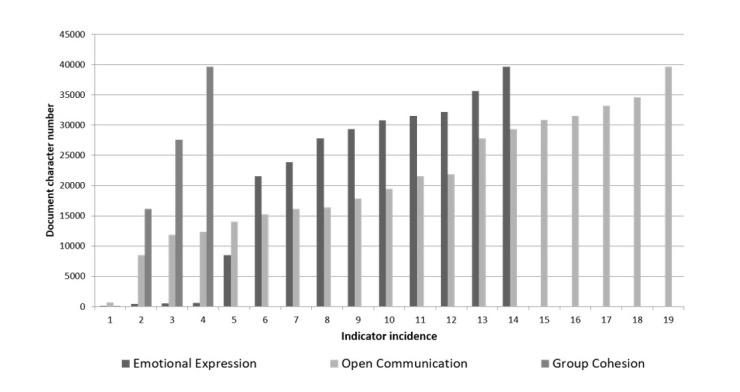
Social presence in synchronous discussion.

**Figure 6:**
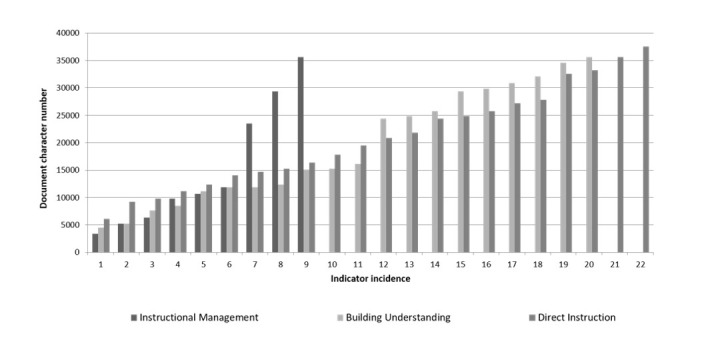
Teaching presence in synchronous discussion.

Figures 4-6 show a breakdown of the instances of the CoI categories within the transcript. The x-axis shows each incidence of coding, against the position (y-axis) within the document (0=start of document, 4000=end). Figure 4 shows the breakdown for cognitive presence. While triggering event, exploration, and integration all appear evenly within the discussion, the resolution category (indicated by application of new ideas, creating solutions, etc) occurred relatively late in the discussion.

Figure 5 shows the breakdown for social presence. Group cohesion occurs early as faculty and students get acquainted, with emotional expression (eg, emotion, autobiographical narrative, humor) occurring toward the middle and end of the hour’s discussion, as they become more relaxed and confident in each other’s presence. The open communication (eg, acknowledgement, risk-free expression) appears to be evenly spread throughout.

Figure 6 displays the breakdown of teaching presence. Instructional management is when the instructor introduces topics. The chart shows a fairly uniform pattern of building understanding and direct instruction throughout the forum.

## Discussion

### Principal Findings

This study appears to be the first such research to use multiple sources of data to describe the characteristics of Community of Inquiry of a whole Web-based course. As such, it is difficult to place the findings in comparison to other literature. Kumar et al [20] are one of the few to have studied a course as a whole, researching asynchronous and synchronous forums. However, they use only a quantitative data source and conclude that social presence was more difficult to foster than cognitive and teaching presences. The university course studied for this paper showed similar levels of presences using the survey tool, although teaching presence was less than “agreed” on the composite score (ie, score <4.0). However, the value of comparing scores of courses as a whole is not clear.

This study does indicate that a CoI is possible across a wide range of learning activities of a Web-based course. It has identified areas of strength and weakness, and as such can aid course developers and others to improve areas of weakness. Specific findings are as follows.

#### Survey

The survey showed that students perceived that a strong CoI was created in the master’s course. The overall composite score of all the items from all respondents was 4.1 (SD 0.46). Cognitive presence was the most strongly perceived, with every item in agreement. The survey asked 12 questions on cognitive presence, three each for triggering event, exploration, integration, and resolution. The results from this study indicate that each of the phases of cognitive presence was well met. However, five of the teaching presence items did not give agreement. It is interesting to see that those consist of three of the first four questions, designed to assess “design & organization,” and also two of the last three questions, designed to assess “direct instruction.” The last two both featured “feedback” in the item, which had been acknowledged within the faculty to have been substandard in the early phases of the course. The design and organization were suboptimal with early teething problems with the administrative aspects of the course. These did improve over the academic year, but the students perceived them to be weaker aspects of the course. High teaching presence has been correlated with high cognitive presence by Akyol and Garrison [17], and Shea and Bidjerano [10] concluded that higher levels of instructor facilitation led to higher cognitive presences. In this study, the survey items that measured “facilitation” all met the criteria for strong agreement, and coupled with the strong cognitive presence, would also support those findings, even though such correlations were not part of the research design.

Social presence has been described in the literature as being crucial to the development of critical discourse in Web-based environments. While social presence alone will not result in deep learning, such learning is extremely unlikely to occur in its absence [10]. The findings from this research show that the students generally felt able to develop group cohesion and were comfortable interacting within the course and among their peers. Garrison in his review of the CoI [11] emphasizes the importance of developing group cohesion as a means of fostering a strong environment that may encourage deep learning, putting less importance on personal relationships and socioemotional presence. The findings of this study reveal strong results for such less important aspects, but Garrison also points out that to develop group cohesion, getting to know each other is an important part early in the process.

#### Asynchronous Discussion Forums

Asynchronous discussion forums are the most dominant form of computer-mediated communication and among the most studied in the literature. This study analyzed the contents of 10 separate discussions, comprising 165 comments and over 18,000 words. The aggregate CoI coding showed that social presence was the most dominant within these discussions, followed by cognitive then teaching presences.

The major criticism of these types of communication has been the lack of social presence and community [21]. The lack of immediacy and dynamic interaction compared to face-to-face communication, as well as the frustrations of posting and reading long messages have all been reported to reduce the engagement and participation of students [22]. This study showed that social interaction is inherently possible in asynchronous forums, as supported by other authors [16,23], and that the creation of a CoI can occur.

The cognitive presence within the discussions was generally higher in this study when instructors participated in the forum (Figure 2), although the last discussion (number 10) had no instructor presence. However, studying only the broad cognitive presence without the breakdown of which element of the presence occurs can either overestimate or underestimate its importance. Evidence of higher levels of thinking (integration and resolution) suggest that more deep and meaningful learning may have occurred [8,24,25]. These studies, however, have stressed how content analysis has generally found that asynchronous discussions develop more instances of lower level thinking. This study would support that finding (Figure 3). The data from analysis of the 10 discussions show that the majority of instances of cognitive presences were coded at the lower cognitive level of triggering event and exploration, with 30% being integration of ideas, but only 7% of instances being at the highest level of resolution.

However, as Garrison has pointed out [8], the CoI occurs only when all three presences occur and that teaching and social presence promote cognitive presence. Figure 1 shows which discussions had the most presences, compared to number of instructor posts. The discussions with most codes (discussion numbers 2, 7, and 8) had more equal proportions of each presence within them, with discussions 2 and 8 having the greatest number of instructor posts. This would suggest that a CoI was created in these posts, but that in these cases the instructor presence appeared to be important in creating that. Discussion number 10 scored most highly for teaching presence; however, there were no posts from an instructor. This was the last thread of the year and suggests the students may have gained more experience and confidence in directing the discussion and sharing their knowledge in the form of direct instruction. Similar findings have been reported by Akyol and Garrison in a study of the CoI [17], who noted increases in teaching presence over time in a course.

#### Synchronous Discussion Forums

These sessions were designed to be the primary source of interaction within the course, allowing students and faculty to get to know each other. Students were encouraged to use webcams for these sessions or audio feeds to increase the interactivity. The forums were places where grievances could be aired and problems discussed with faculty members. They typically consisted of casual opening exchanges, as well as the learning experiences from what was hoped to be a CoI.

The findings of this study suggest that these sessions did provide such a community. The content analysis of the transcript of one such forum shows 51 instances of teaching presence, 47 instances of cognitive presence, and 37 instances of social presence in a 1-hour session. Despite the ability of the forum to develop social interaction, the social presence was lower than the other two presences of the CoI framework. It is possible that this lower presence was because the forum that was chosen for analysis was in the middle of the course and that students and faculty were already acquainted. However, one might expect more “banter” as characters become better known.

The breakdown of CoI categories in the transcribed forum showed that the elements of practical inquiry (ie, triggering, exploration, integration, and resolution) were similarly expressed within the cognitive domain, whereas the emotional expression and open communication were dominant in the social presence domain. Direct instruction from the faculty member and his ability to build understanding were also high on the teaching presence front.

These findings correlate with results from the literature. Groen et al [26] comment on the ability of casual chat to build a sense of community, and that is reflected by emotional expression being the dominant coding category in this study’s content analysis (Figure 5). The steady presence of the open communication category throughout the discussion also represents development of the community within the session, while the strong cognitive presence indicates meaningful learning could have occurred.

The potential of deeper learning is supported by the cognitive breakdown (Figure 4). The exploration of themes tended to occur earlier in the discussion than integration and resolution, suggesting that as the forum progressed the students were more capable of higher levels of thinking as their understanding of the topics increased.

Teaching presence was strong in the synchronous discussion (Figure 6). This raises a possible concern. The presence of a dominant teacher runs the risk of the session developing into a teacher-centered activity, reducing the learning potential from a constructivist perspective [27]. The content analysis of the transcribed forum had a heavy direct instruction component, and 117 of the 324 posts were from the faculty member. However, there is no evidence from this study that information overload or distraction was a problem in this course, as Hiltz and Turoff [28] have warned against.

### Strengths and Limitations

There are several limitations to this study. The setting of a single graduate-level course meant that the number of participants available were small. Only 22 students were initially enrolled in the course, and two withdrew early in the academic year resulting in 20 students eligible for the study. The course was also in its first year of existence and thus experienced difficulties at the start. This was quickly recognized by the faculty and administrative staff but resulted in suboptimal administrative tasks and communication, delays in feedback processes, and early technology difficulties. This may have influenced some of the study results. Toward the middle and end of the year, these processes were tightened and strategies to ensure timely feedback and clarity in organization were instigated.

The master’s program in this research was in a health care discipline, and the area of study a highly niche topic with no such comparable course available worldwide either in topic or delivery. Therefore, it is hard to know how transferrable these findings may be to other disciplines or courses.

The CoI survey was sent to students only once approximately 6 months after the end of the year in question. An earlier mid-course survey combined with the end of year survey would have enabled comparison of how a CoI changed over time and whether the problems listed above may have altered the findings.

Time and resource restraints meant that only a selection of discussion forums was analyzed. Therefore, it is unclear whether the results are generalizable. This was mitigated in part by choosing a synchronous forum in the middle of the year, with no prior knowledge of content or participants. A range of asynchronous forums across the year were also chosen to try to obtain a representative sample of the course.

However, the research also has strengths. The relatively small number of participants enabled an in-depth examination of the study question. Larger sample sizes may have resulted in an amount of data that would not be possible for a single researcher to analyze. Although the course was a health care discipline, the participants were inter-professional graduates. This resulted in differing perspectives and backgrounds giving the researcher more enriched data than may have been available if only physicians or nurses had been enrolled.

Areas for future research include repeating the study in future years to compare how CoI may change as the course becomes more mature and as faculty and administration are more familiar with Web-based learning and reacting to feedback. A larger study would allow more content analysis of discussion forums. How CoI changes over time could be studied by analyzing both synchronous and asynchronous forums in a more structured manner at set times of the year.

### Conclusion

The results of this study show that a Community of Inquiry is possible in a Web-based master’s program. The significance of this study is in its methodology. It has set out to explore the CoI not only of a course in its entirety, but also within some of its constituent parts. Analysis of the CoI survey has shown global trends over the year. The content analysis provided rich information that would not have been evident from just the survey and highlighted areas of pedagogical strengths and weaknesses, which can improve the CoI presence of the course if addressed. These results would suggest that strong learning opportunities are entirely possible in Web-based courses.

## Abbreviations

CoI: Community of Inquiry
MSc: Master of Science
